# A Novel Single-Excitation Capacitive Angular Position Sensor Design

**DOI:** 10.3390/s16081196

**Published:** 2016-07-29

**Authors:** Bo Hou, Bin Zhou, Mingliang Song, Zhihui Lin, Rong Zhang

**Affiliations:** Engineering Research Center for Navigation Technology, Department of Precision Instrument, Tsinghua University, Beijing 100084, China; houb15@mails.tsinghua.edu.cn (B.H.); songmingliang1@gmail.com (M.S.); linzhihui1995@163.com (Z.L.)

**Keywords:** angular position sensor, capacitance sensitive, single-excitation, sinusoidal circular electrodes, amplitude modulation

## Abstract

This paper presents a high-precision capacitive angular position sensor (CAPS). The CAPS is designed to be excited by a single voltage to eliminate the matching errors of multi-excitations, and it is mainly composed of excitation electrodes, coupling electrodes, petal-form sensitive electrodes and a set of collection electrodes. A sinusoidal voltage is applied on the excitation electrodes, then the voltage couples to the coupling electrodes and sensitive electrodes without contact. The sensitive electrodes together with the set of collection electrodes encode the angular position to amplitude-modulated signals, and in order to increase the scale factor, the sensitive electrodes are patterned in the shape of petal-form sinusoidal circles. By utilizing a resolver demodulation method, the amplitude-modulated signals are digitally decoded to get the angular position. A prototype of the CAPS is fabricated and tested. The measurement results show that the accuracy of the sensor is 0.0036°, the resolution is 0.0009° and the nonlinearity over the full range is 0.008° (after compensation), indicating that the CAPS has great potential to be applied in high-precision applications with a low cost.

## 1. Introduction

Angular position and rate information are useful in a wide variety of applications, such as automation control systems and industrial manufacturing. With the development of the intelligence industry, the demand for high-precision, high-reliability, low-cost and environmentally-robust angular position sensors has substantially increased [1,2,3].

Optical angular sensors and electromagnetic resolvers are widely-used noncontact angular displacement sensors [4]. Optical sensors can realize angular position measurement resolution up to several arcsecs after interpolation; however, their performance decays in harsh environments, such as those with mechanical vibration, extreme temperature variations or dust pollution. Electromagnetic resolvers perform better in harsh environments, but they do not provide sufficient resolution and accuracy [5,6]. Furthermore, both optical encoders and electromagnetic resolvers are relatively expensive and are commonly used in high-end application scenarios.

Capacitive angular sensors are expected to provide some significant advantages over optical encoders and resolvers [4,7,8]. Compared to electromagnetic resolvers, the capacitive angular sensor has the unique qualities of low cost and simple structure, since the main components of the capacitive angular sensors are electrode plates, which are made from standard printed circuit board (PCB) technology [2,9]. In spite of its sensitivity to industrial pollution, such as moisture and oils, which would be prevented by a good shielding, the capacitive encoder still tends to be a critical device, as it not only combines good robustness with high accuracy, but also realizes low cost, a simple structure, and low power consumption.

In recent years, there has been a great deal of research on capacitive angular sensors [10]. Brasseur has presented a variety of capacitive encoders that meet a variety of unique application demands [11,12,13]. The capacitive encoders discussed by Brasseur require that four different excitation signals should be applied. These four excitations and detecting periods make the measurement time excessive, so these encoders can only be used for detection at very low speeds (<5°/s) [13]. Japanese scholar Fumitaka Kimura proposed a resolver-compatible capacitive rotary position sensor with a simple and versatile sensitivity structure design with a nonlinearity error of ±4°, but its accuracy and linearity need further improvement [8,14,15]. Despite its relatively low precision and low linearity, the capacitive rotary resolver has great significance in terms of the demodulation of capacitive signals. Zheng et al., inspired by the element structure established by Israeli scientists [16,17,18], developed a modem capacitance encoder that is highly accurate and highly dynamic. Its nonlinearity is ±0.4°, and the precision is better than 0.006° [5]. However, its demodulation circuit is complex, and its accuracy is limited by the matching errors between the two excitation signals on amplitude and phase. In conclusion, the real-time angle tracking performance is poor, and the accuracy or nonlinearity needs enhancement. Besides, the accuracy of capacitive angular sensors is limited by the matching errors of multi-excitations on the amplitude and phase. Therefore, efforts should be made to increase the scale factor, to eliminate the matching errors of multi-excitations and to suppress the nonlinear characteristics in future research.

In this paper, we present a novel single-excitation capacitive sensor (capacitive angular position sensor (CAPS)) whose structure is similar to the one in [5], but with a different sensing mechanism and signal processing. Based on the designed petal-form sensitive electrodes and a set of collection electrodes, the position of the rotor is encoded into an excitation sinusoidal signal. The sinusoidal signal is modulated to sine and cosine by the rotor position. After calculating the relationship of the sine and cosine signals by a resolver chip, the angular position is obtained. The CAPS is designed to be excited by a single voltage to eliminate the matching errors of multi-excitations. Further, to increase the scale factor, the sensitive electrode of CAPS is patterned in the shape of petal-form sinusoidal circles.

This paper is organized as following: In Section 2, the sensing element, working principle and signal demodulation are discussed. In Section 3, the prototype and the measurement setup are given. Afterwards, the experiment results are presented and discussed in Section 4. Section 5 provides a brief summary and conclusion.

## 2. Basic Principle and Design

### 2.1. Sensing Element

**Figure 1:**
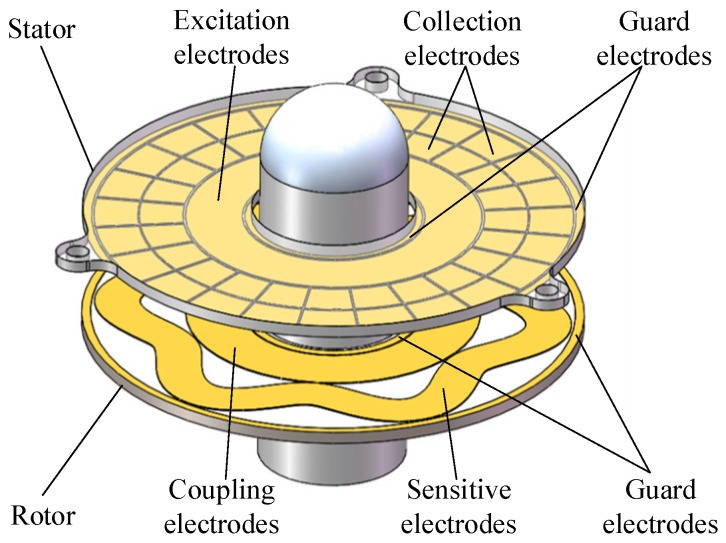
Structural model of the capacitive angular position sensor (CAPS).

The basic sensing element of the capacitive angular position sensor is composed of a stator and a rotor [19], both of which are fabricated from standard PCB technology, as shown in Figure 1. The stator is designed to be fixed on a metal housing through screw holes, and the rotor is connected to a shaft that rotates with a moving element. One side of the rotor, facing the stator, is covered with sensitive electrodes and coupling electrodes, and one side of the stator, which is facing the rotor, is covered with a set of collection electrodes and excitation electrodes. The sensitive electrode is a petal-form sinusoidal circle, which overlaps the collection electrodes, and the coupling electrodes are connected with sensitive electrodes and are located inside of it. The collection electrodes are sector areas arranged in the circumference direction to form two circular rings, and the excitation electrodes are located inside the circular ring. Surrounding the four electrodes, circular guarding electrodes are applied to reduce the influence of external interference.

### 2.2. Measurement Principle

**Figure 2:**
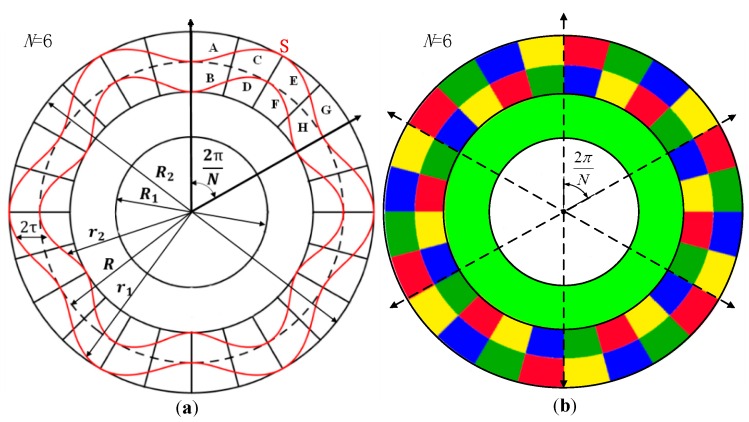
(**a**) Schematic view of the capacitive angular position sensor; (**b**) schematic diagram of the stator, in which the same color areas are connected.

The schematic view of this capacitive sensor is shown in Figure 2a. The petal-form sensitive electrodes indicated by S are sine wave shapes in polar coordinates with *N* cycles. Every sine petal faces eight collection electrodes, which are indicated by A, B, C, D, E, F, G and H, respectively. The eight collection electrodes are divided into four groups, and the groups A and F, B and E, C and H, D and G are interconnected to form four electrode sets. The four electrode sets are placed alternately and repeatedly with *N* cycles, which is the same as the sensitive electrode. As shown in Figure 2b, the same color areas of the collection electrodes are connected to form four electrode sets. This structure is less sensitive to mechanical non-idealities, such as the eccentricity and the obliqueness of the rotor, because these non-idealities are averaged over the multiple cycles [20,21].

To detect the varying of capacitances between the sensitive electrode and the collection electrodes, a single sinusoidal signal with high frequency is applied on the excitation electrode [22,23]. Being faced with the excitation electrode, the coupling electrodes can give rise to a coupled signal $U_{s}$. The coupling electrodes and the sensitive electrodes are connected, which means that the coupled signal conducts to the sensitive electrode. As the collection electrodes overlap the sensitive electrodes, the signal $U_{s}$ is coupled back to the collection electrodes. On the set of collection electrodes, four amplitude-modulated signals encoded by the rotor rotation angle are detected. The high-frequency excitation signal applied to the excitation electrodes of the stator is expressed as follows:

(1) $$U_{e}=A_{e}\cdot \sin(wt)$$

where w is the frequency of excitation voltages, $A_{e}$ is the amplitude and t is time.

**Figure 3:**
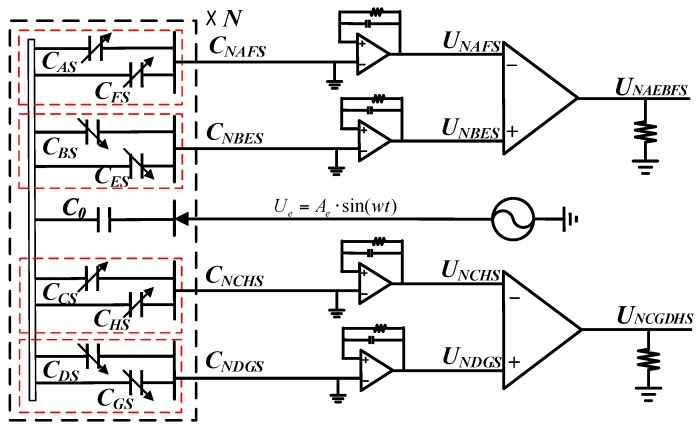
Circuit model diagram of CAPS. Capacitances $C_{AS}$, $C_{BS}$, $C_{CS}$, $C_{DS}$, $C_{ES}$, $C_{FS}$, $C_{GS}$ and $C_{HS}$ are formed by the sensitive electrode and the collection electrodes in each sine petal cycles shown in Figure 2a. Capacitances $C_{NAFS}$, $C_{NBES}$, $C_{NCHS}$, $C_{NDGS}$ are formed by the eight capacitances in the complete sensitive electrode (as shown in Figure 2b, the same color areas of the collection electrodes are connected to form $C_{NAFS}$, $C_{NBES}$, $C_{NCHS}$, $C_{NDGS}$).

Figure 3 shows a simple circuit model of the CAPS without considering the parasitic capacitances [8,9,24]. The excitation electrodes together with coupling electrodes form the unchanged excitation capacitances $C_{0}$ (neglecting mechanical non-idealities). Each sine petal cycle of the sensitive electrode together with the collection electrodes form $C_{AS}$, $C_{BS}$, $C_{CS}$, $C_{DS}$, $C_{ES}$, $C_{FS}$, $C_{GS}$ and $C_{HS}$, which will change with the rotor rotation angle ϕ. The eight capacitances are connected to form four measurement capacitances, $C_{NAFS}$, $C_{NBES}$, $C_{NCHS}$, $C_{NDGS}$. The changes of the four measurement capacitances are converted to voltage through four charge amplifiers (C-V conversion modules), respectively. In addition, two differential amplifiers for picking up the converted signals keep in step with the charge amplifier.

The capacitance of a parallel-plate capacitor is determined by the geometry of the conductive plates and the dielectric properties of the insulator between the plates. The capacitances $C_{AS}$, $C_{BS}$, $C_{CS}$, $C_{DS}$, $C_{ES}$, $C_{FS}$, $C_{GS}$ and $C_{HS}$ can be given by $C=\varepsilon_{0}S/d$; *d* is the distance between the stator and rotor; and *S* is the area of overlap of collection electrodes and the sensitive electrode. Capacitance is proportional to *S*; therefore, varying of the area of overlap between collection electrodes and the sensitive electrode is crucial to the signal modulation.

The sensitive electrode is in sine wave shapes in polar coordinates with *N* cycles. The contour line of the sensitive electrode can be expressed as:

(2) $$\left\{ \begin{array}{l} r_{1}=R+\tau (\sin(N*\phi )+1)\\ r_{2}=R+\tau (\sin(N*\phi )-1) \end{array}\right.$$

where $r_{1}$ represents the inner contour line and $r_{2}$ the outer contour line. R is the radius of the circle on which the sine wave is wrapped; *τ* is the amplitude of the sine wave; *N* is a positive integer; ϕ represents the rotation angle relative to the X-axis of the polar coordinate and is also the mechanical angle between the rotor and stator.

According to the area formula of a polar curve $S=1/2\int r^{2}d\theta$, the overlapping areas between each sinusoidal cycle of the sensitive electrodes and collection electrodes is calculated as follows:

(3) $$\left\{ \begin{array}{l} S_{AS}=\int_{(-\pi /4+\theta )/N}^{(\pi /4+\theta )/N}\frac{1}{2}({r_{1}}^{2}-R^{2})d\theta \\ S_{BS}=\int_{(-\pi /4+\theta )/N}^{(\pi /4+\theta )/N}\frac{1}{2}(R^{2}-{r_{2}}^{2})d\theta \\ S_{CS}=\int_{(-\pi /4+\pi +\theta )/N}^{(\pi /4+\pi +\theta )/N}\frac{1}{2}({r_{1}}^{2}-R^{2})d\theta \\ S_{DS}=\int_{(-\pi /4+\pi +\theta )/N}^{(\pi /4+\pi +\theta )/N}\frac{1}{2}(R^{2}-{r_{2}}^{2})d\theta \end{array}\right.$$

(4) $$\left\{ \begin{array}{l} S_{ES}=\int_{(\pi /4+\theta )/N}^{(3\pi /4+\theta )/N}\frac{1}{2}({r_{2}}^{2}-R^{2})d\theta \\ S_{FS}=\int_{(\pi /4+\theta )/N}^{(3\pi /4+\theta )/N}\frac{1}{2}(R^{2}-{r_{2}}^{2})d\theta \\ S_{GS}=\int_{(\pi /4+\pi +\theta )/N}^{(3\pi /4+\pi +\theta )/N}\frac{1}{2}({r_{1}}^{2}-R^{2})d\theta \\ S_{HS}=\int_{(\pi /4+\pi +\theta )/N}^{(3\pi /4+\pi +\theta )/N}\frac{1}{2}(R^{2}-{r_{2}}^{2})d\theta \end{array}\right.$$

where θ is the electric angle and θ=N⋅ϕ, which means the electric angle is *N*-times the mechanical angle. Equations (5) and (6), corresponding to the integration of Equations (3) and (4) accordingly, show that there are direct current (DC) components, cos(θ) and sin(θ) components and cos(2θ) components in the equations.

(5) $$\begin{array}{l} \left\{\begin{array}{l} S_{AS}=[(3\pi +8\sqrt{2}\cos(\theta )+2\cos(2\theta ))\cdot \tau +4(\pi +2\sqrt{2}\cos(\theta ))\cdot R]\cdot \tau /8N\\ S_{FS}=-[(3\pi +8\sqrt{2}\cos(\theta )+2\cos(2\theta ))\cdot \tau -4(\pi +2\sqrt{2}\cos(\theta ))\cdot R]\cdot \tau /8N \end{array}\right\}\\ \left\{\begin{array}{l} S_{BS}=[(3\pi +8\sqrt{2}\cos(\theta )+2\cos(2\theta ))\cdot \tau +4(\pi -2\sqrt{2}\cos(\theta ))\cdot R]\cdot \tau /8N\\ S_{ES}=-[(3\pi +8\sqrt{2}\cos(\theta )+2\cos(2\theta ))\cdot \tau -4(\pi -2\sqrt{2}\cos(\theta ))\cdot R]\cdot \tau /8N \end{array}\right\} \end{array}$$

(6) $$\begin{array}{l} \left\{\begin{array}{l} S_{CS}=[(3\pi +8\sqrt{2}\sin(\theta )+2\cos(2\theta ))\cdot \tau +4(\pi +2\sqrt{2}\sin(\theta ))\cdot R]\cdot \tau /8N\\ S_{HS}=-[(3\pi +8\sqrt{2}\sin(\theta )+2\cos(2\theta ))\cdot \tau -4(\pi +2\sqrt{2}\sin(\theta ))\cdot R]\cdot \tau /8N \end{array}\right\}\\ \left\{\begin{array}{l} S_{DS}=[(3\pi +8\sqrt{2}\sin(\theta )+2\cos(2\theta ))\cdot \tau +4(\pi -2\sqrt{2}\sin(\theta ))\cdot R]\cdot \tau /8N\\ S_{GS}=-[(3\pi +8\sqrt{2}\sin(\theta )+2\cos(2\theta ))\cdot \tau -4(\pi -2\sqrt{2}\sin(\theta ))\cdot R]\cdot \tau /8N \end{array}\right\} \end{array}$$

As stated before, the eight collection electrodes are divided into four groups, and in each group, the electrodes are connected with each other. The connected area change relationships are expressed as follows:

(7) $$\left\{ \begin{array}{l} S_{NAFS}=N(S_{AS}+S_{FS})=(\pi +2\sqrt{2}\cos(\theta ))\cdot R]\cdot \tau =(\pi +2\sqrt{2}\cos(N\phi ))R]\cdot \tau \\ S_{NBES}=N(S_{BS}+S_{ES})=(\pi -2\sqrt{2}\cos(\theta ))\cdot R]\cdot \tau =(\pi -2\sqrt{2}\cos(N\phi ))R]\cdot \tau \\ S_{NCHS}=N(S_{CS}+S_{HS})=(\pi +2\sqrt{2}\sin(\theta ))\cdot R]\cdot \tau =(\pi +2\sqrt{2}\sin(N\phi ))R]\cdot \tau \\ S_{NDGS}=N(S_{DS}+S_{GS})=(\pi -2\sqrt{2}\sin(\theta ))\cdot R]\cdot \tau =(\pi -2\sqrt{2}\sin(N\phi ))R]\cdot \tau \end{array}\right.$$

which will be used later.

Based on the circuit model diagram of CAPS, the coupled signal $U_{s}$ generated on coupling electrodes and the sensitive electrode can be expressed as:

(8) $$U_{S}=kU_{e}=kA_{e}\cdot \sin(wt)$$

where *k* is the coupling coefficient and $k=C_{0}/(C_{0}+C_{NAFS}+C_{NBES}+C_{NCHS}+C_{NDGS})$.

The charges on the collection electrodes can be given by multiplying the capacitances, $C_{NAFS}$, $C_{NBES}$, $C_{NCHS}$ and $C_{NDGS}$, with the generated excitation voltages $U_{s}$ on coupling electrodes, respectively, and can be expressed as:

(9) $$\left\{ \begin{array}{l} Q_{NAFS}=\frac{1}{d}\varepsilon \cdot (\pi +2\sqrt{2}\cos(N\phi ))\cdot R]\cdot \tau kA_{e}\sin(wt)=(D+K\cos(N\phi ))\sin(wt)\\ Q_{NBES}=\frac{1}{d}\varepsilon \cdot (\pi -2\sqrt{2}\cos(N\phi ))\cdot R]\cdot \tau kA_{e}\sin(wt)=(D-K\cos(N\phi ))\sin(wt)\\ Q_{NCHS}=\frac{1}{d}\varepsilon \cdot (\pi +2\sqrt{2}\sin(N\phi ))\cdot R]\cdot \tau kA_{e}\sin(wt)=(D+K\sin(N\phi ))\sin(wt)\\ Q_{NDGS}=\frac{1}{d}\varepsilon \cdot (\pi -2\sqrt{2}\sin(N\phi ))\cdot R]\cdot \tau kA_{e}\sin(wt)=(D-K\sin(N\phi ))\sin(wt) \end{array}\right.$$

where $D=\varepsilon \tau \pi k\cdot A_{e}/d$ is the DC component and $K=2\sqrt{2}\varepsilon \tau k\cdot A_{e}\cdot R/d$ is the amplitude of the charge signal on the collection electrodes.

Next, four charge amplifiers are applied to convert capacitance changes to voltage and two differential amplifiers are applied to eliminate the common element. Via conversion and subtraction, the common element in Equation (9) can be counteracted, and the equation can be simplified as:

(10) $$U_{NAEBFS}=G_{c}\cdot G_{a}\cdot (Q_{NAFS}-Q_{NBES})=2G_{c}\cdot G_{d}K\cos(N\phi )\sin(wt)+n(t)=U_{0}\cos(\theta )\sin(wt)+n(t)$$

(11) $$U_{NCGDHS}=G_{c}\cdot G_{a}\cdot (Q_{NCGS}-Q_{NDHS})=2G_{c}\cdot G_{a}K\sin(N\phi )\sin(wt)+n(t)=U_{0}\sin(\theta )\sin(wt)+n(t)$$

where $G_{c}$ is the gain of the charge amplifier, $G_{a}$ is the gain of the differential amplifier, $U_{0}=2G_{c}G_{d}K=4\sqrt{2}\varepsilon k\cdot A_{e}G_{c}G_{d}\cdot \tau R/d$ is the amplitude of output signals, θ=N⋅ϕ is the electric angle and n(t) is the noise of each signal. As shown in Equations (10) and (11), the rotation mechanical angle ϕ is encoded to the signal $U_{NAEBF}$ and $U_{NCGDH}$ by one excitation signal sin(wt) based on the designed sensitive structure.

From Equations (10) and (11), it can be concluded that the position of the rotor is encoded into the amplitude of the signals $U_{NAEBF}$ and $U_{NCGDH}$. When the number of petals increases, the amplitude of the signals $U_{NAEBF}$ and $U_{NCGDH}$ does not decrease, and the mechanical angle can be further subdivided. Therefore, the scale factor can be improved considerably with the increase of the petal-shape number *N*. The greater the number of petals on sensitive electrodes, the lager the scale factor is. However, *N* could not be increased infinitely, as the manufacturing error grows with N. In the article, *N* is set to be six.

### 2.3. Signal Demodulation

**Figure 4:**
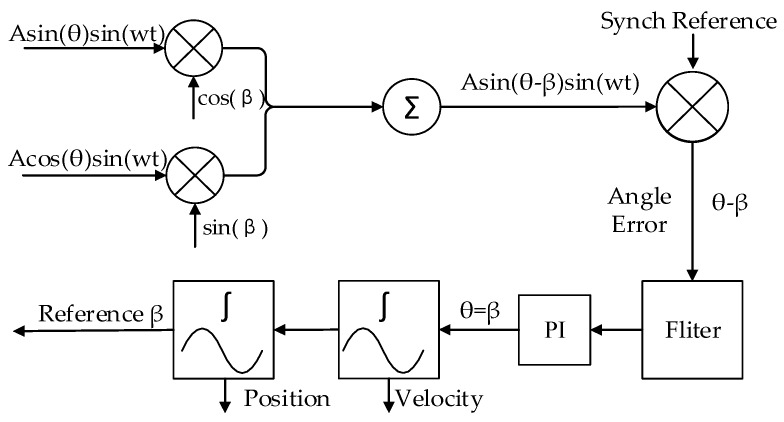
Block diagram of electric angle signal demodulation.

As shown in Equations (10) and (11), the capacitive angular position sensor output two amplitude modulated signals, which can be demodulated by the traditional amplitude demodulation techniques of resolvers [25,26]. The block diagram of the demodulation is shown in Figure 4. An angle β is produced in the resolver chip to track the shaft angle θ. β is fed back and compared to the input angle θ continually, and when the resulting error between the two is zero, the produced angle β is correctly tracking the input angle θ. To measure the error, $U_{NAEBFS}$ is multiplied by cos(β) and $U_{NCGDHS}$ is multiplied by sin(β) to give:

(12) $$E_{1}=U_{0}\cos(\beta )\cdot \sin(\theta )\cdot \sin(wt)$$

(13) $$E_{2}=U_{0}\sin(\beta )\cdot \cos(\theta )\cdot \sin(wt)$$

The difference between two signals is:

(14) $$e=U_{0}\;\sin wt\cdot (\sin\beta \cos\theta -\cos\beta \sin\theta )$$

This signal is demodulated using the internally-generated synthetic reference,

(15) $$e=U_{0}(\sin\theta \cos\beta -\cos\theta \sin\beta )=U_{0}\sin(\theta -\beta )(1-\cos(2wt))/2$$

A phase-sensitive demodulator, some integrators, a PI controller and a compensation filter constitute a closed-loop system that seeks to null the error signal. When the loop is accomplished, β equals the resolver angle θ within the rated accuracy of the converter.

## 3. Prototype and Measurement Setup

### 3.1. Geometrical Dimensions of the Prototype

**Figure 5:**
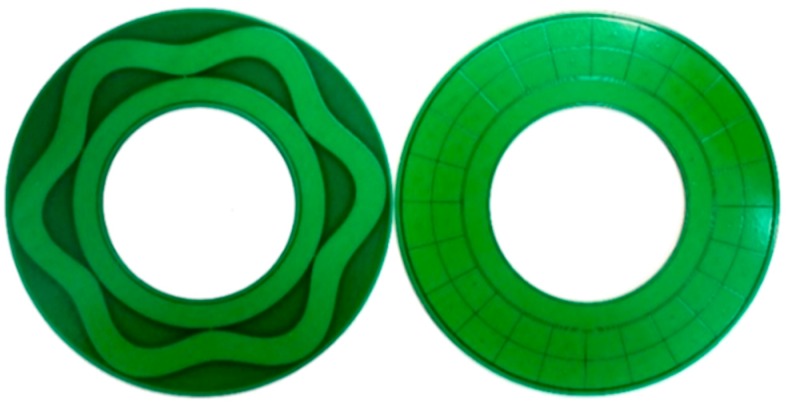
Sensitive structures of the prototype of CAPS.

To verify the sensing principle, a prototype has been fabricated. Figure 5 shows the photograph of the stator and the rotor plates, both of which are fabricated by standard PCB technology with a diameter of 54 mm. Other geometrical dimensions of the prototype are shown in Table 1. The shielding housing is absent in the prototype. Hence, the rotor is directly mounted on the turntable, and the stator is fixed on the supports of the mounting system that is used to maintain the distance between the rotor and the stator. Surrounding the collection electrodes and sensitive electrodes, circular guarding electrodes are applied to reduce the influence of external interference. Additionally, a large area of copper connected to the ground is used to suppress the effect of the parasitic capacitance.

### 3.2. Signal Processing of the Prototype

**Figure 6:**
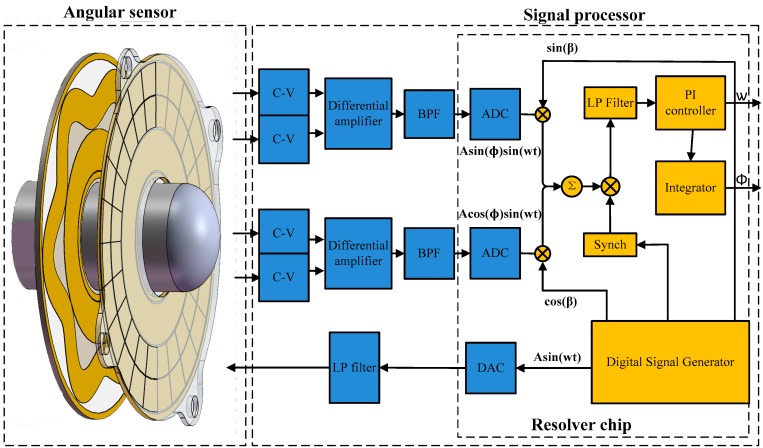
Signal processing architecture of the capacitive angular position sensor.

The signal processing architecture of the prototype is shown in Figure 6. As the key component, the resolver decoder chip (AD2S1210) realizes the most signal processing operations, including generating the excitation voltage and demodulation reference signals, calculating some integral, converting the electrical angle to mechanical angle and sending the digital angular position to the USB interface. All of them are more easily realized in the resolver decoder chip compared to conventional analog circuits. Moreover, the digital demodulation through the integrated chip is more accurate than the discrete components.

The digital signal generator works by first storing a period of sampled sine wave in onboard memory and then outputting these digital values in a predetermined sequence. Through four 16-bit digital to analog converters (DAC), the digital excitation signal is converted to analog signals with the frequency of 10 kHz, and thereafter, it is applied on excitation electrodes. Four capacitance changes are generated on the collection electrodes. After being converted by the C-V conversion modules and differential amplifiers, two output voltages of the CAPS are obtained and then passed through third-order Butterworth band pass filters (BPF) with the center frequency of ω to eliminate the noise n(t). Next, the voltages are sampled by a 16-bit analog to digital converter (ADC) at the frequency of 400 kHz and are then fed into the resolver decoder chip to implement the quadrature demodulation digitally, which is described in Figure 4. Considering the carrier frequency of 10 kHz and the max frequency shift of the output voltage of ±120 Hz determined by the max rotating speed of 1200 r/min, the center frequency of the BPF is set at 10 kHz with a bandwidth of 1 kHz, and the cutoff frequency of the low pass filters LPF is set at 1 kHz.

### 3.3. Experimental Platform

**Figure 7:**
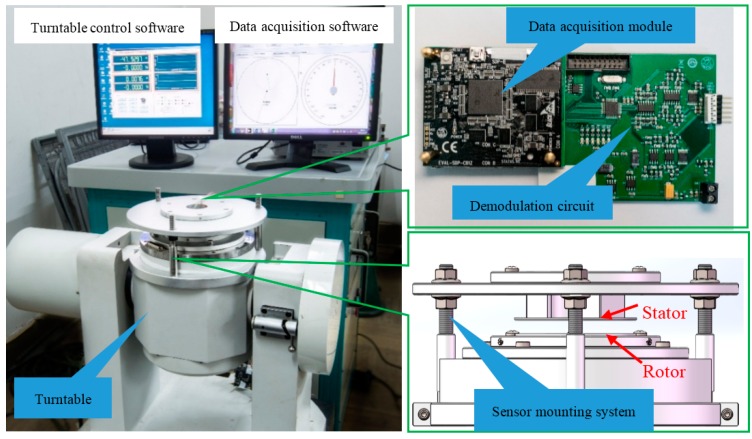
Experimental platform for testing prototype, a high-precision turntable, a demodulation circuit, data acquisition module and a sensor mounting system. The rotor was mounted on the shaft of the turntable, and the stator was fixed on the supports of the mounting system.

Figure 7 shows the experimental setup for testing the characteristics of the prototype. The setup consists of a high-precision turntable (Aviation Industry Co., Beijing, China), a sensor mounting system, a demodulation circuit and a data acquisition module (Analog Devices Co., Austin, TX, USA). The turntable, with an accuracy of 0.0001°, was used to change the angular position and angular speed. The sensor mounting system was designed to connect the turntable with the rotor and to adjust the concentricity and parallelism between the rotor and stator. Due to the absence of housing, the rotor was directly mounted on the shaft of the turntable, and the stator was fixed on the supports of the mounting system. The distance between the rotor and the stator can be also adjusted through the sensor mounting system during experimentation. The high-precision turntable measured the angular position of the rotor as the reference. When the rotor stops at a certain place, we can test the precision of the encoder, and when the rotor is rotating at a certain speed, we can test the speed and the dynamic nonlinearity over a full range. The angular position was sent to a computer through the data acquisition module.

## 4. Results and Discussion

### 4.1. Measurement Results

#### 4.1.1. Signals

**Figure 8:**
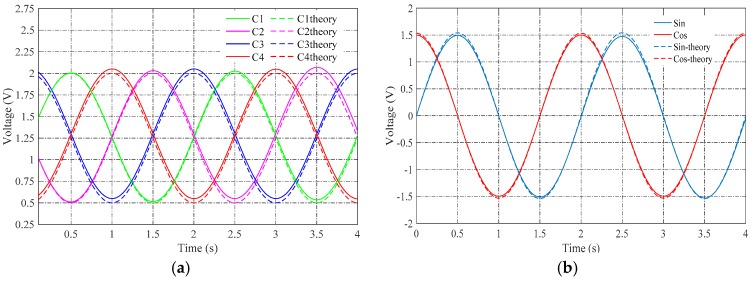
(**a**) Measurement results of capacitance changes; (**b**) measured voltages of sin and cos.

The voltages corresponding to the capacitances $C_{NAFS}$, $C_{NBES}$, $C_{NCHS}$ and $C_{NDGS}$ were measured on the charge amplifiers. The voltages are shown in Figure 8a. They should change as sinusoidal waves as Equation (9). However, a part of them form a non-sinusoidal wave. This was probably caused by the manufacturing error on the electrodes and the install error between rotor and stator.

The measured voltages corresponding to Equations (10) and (11) are plotted in Figure 8b. The experimental results showed amplitude-modulated signals corresponding to theoretical arithmetic; and the envelopes of the two signals showed a 90-degree phase shift, which is the same as the resolver.

#### 4.1.2. Precision

**Figure 9:**
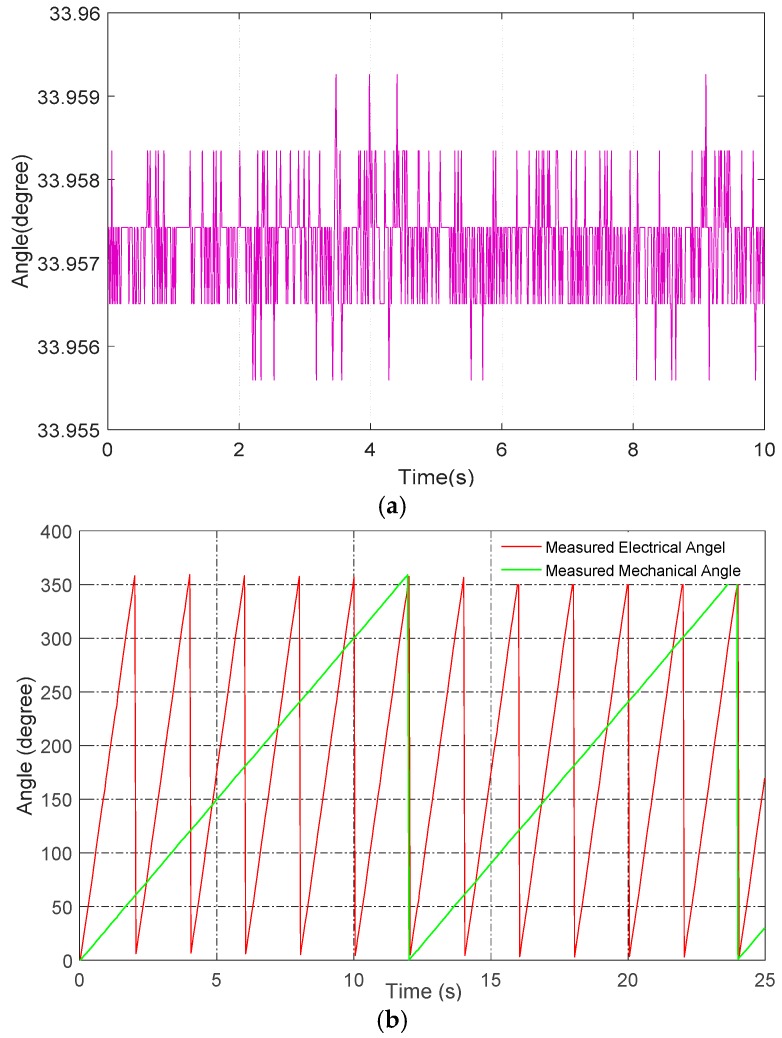
(**a**) Measured angular position when the rotor is stationary; (**b**) measured electrical angle and mechanical angle.

Figure 9a shows the measured angular position when the rotor was stationary. From the fluctuation extent of the angular position, it was concluded that the stationary precision of the capacitive encoder is <0.0036°. Any random fluctuation of the angular position was likely caused by external interference and electrical noise, due to the absence of housing or any other interference-shielding measures. If better shielding measures had been adopted, the precision would likely have been higher. The resolution is clearly visible in Figure 9a, and it is one LSB of the resolver decoder chip, which is roughly 0.0009°.

Further, the dynamic range of CAPS was measured. As shown in Figure 9b, when the turntable rotates at 30°/s, it takes 2 s to sample one cycle of the result corresponding to the rotating speed of 30°/s. The measured results are very consistent with the theoretical calculations.

#### 4.1.3. Linearity

**Figure 10:**
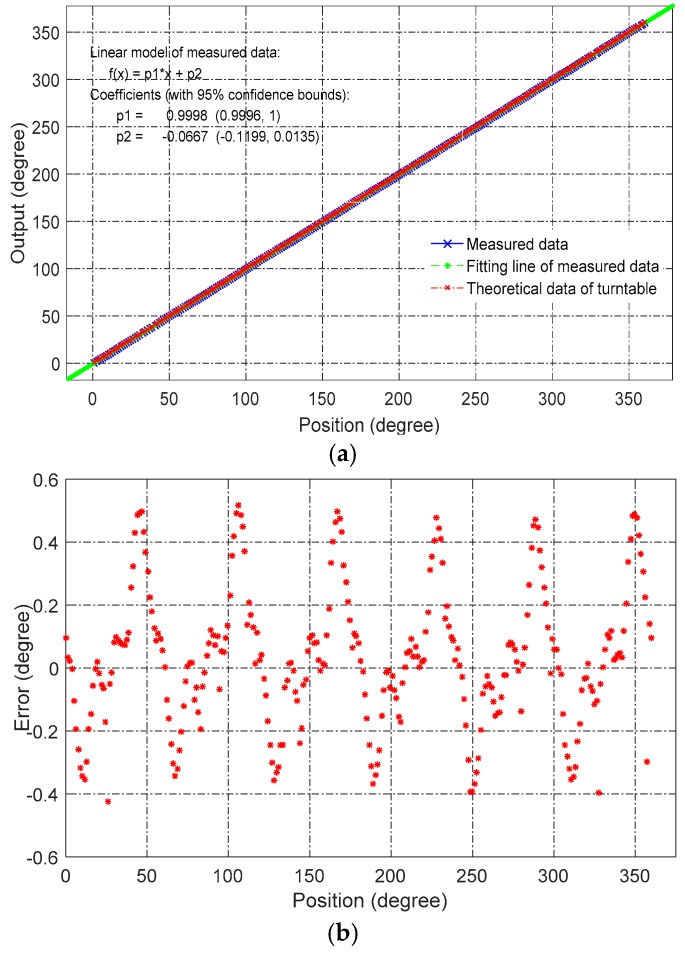
(**a**) Measured angular position and its best-fitting line; (**b**) nonlinearity over a full range with respect to the best-fitting line.

To analyze the dynamic nonlinearity error, we imported the sampled data into MATLAB software. Figure 10a shows the output of the prototype sensor, which was plotted against the reference angle of the turntable. Figure 10b shows the nonlinear error of Figure 10a, where the maximum nonlinearity is ±0.5°.

**Figure 11:**
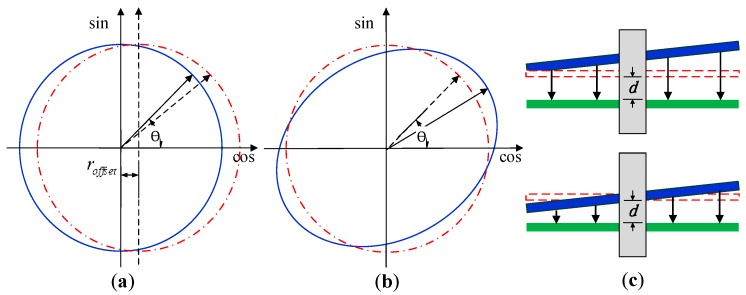
Typical manufacturing error and installation error. (**a**) Rotor and stator do not overlap appropriately; (**b**) rotor and stator are not manufactured as perfect circles; (**c**) rotor and stator are not parallel when measured on the turntable. In the experiment, the designed sensor mounting system can reduce the installation error, but cannot eliminate it.

The nonlinear error was probably caused by four factors:

- Typical manufacturing error and installation error, as shown in Figure 11 (which are periodic and can be compensated).
- Deviations of the capacitance coefficients between the theoretical and real values (which could be improved by modifying the electrode shapes).
- Interference caused by stray capacitors, which formed between cables (which can be reduced by rearranging the cables).
- Soldering on the stator (which could be eliminated by increasing the thickness of the stator).

**Figure 12:**
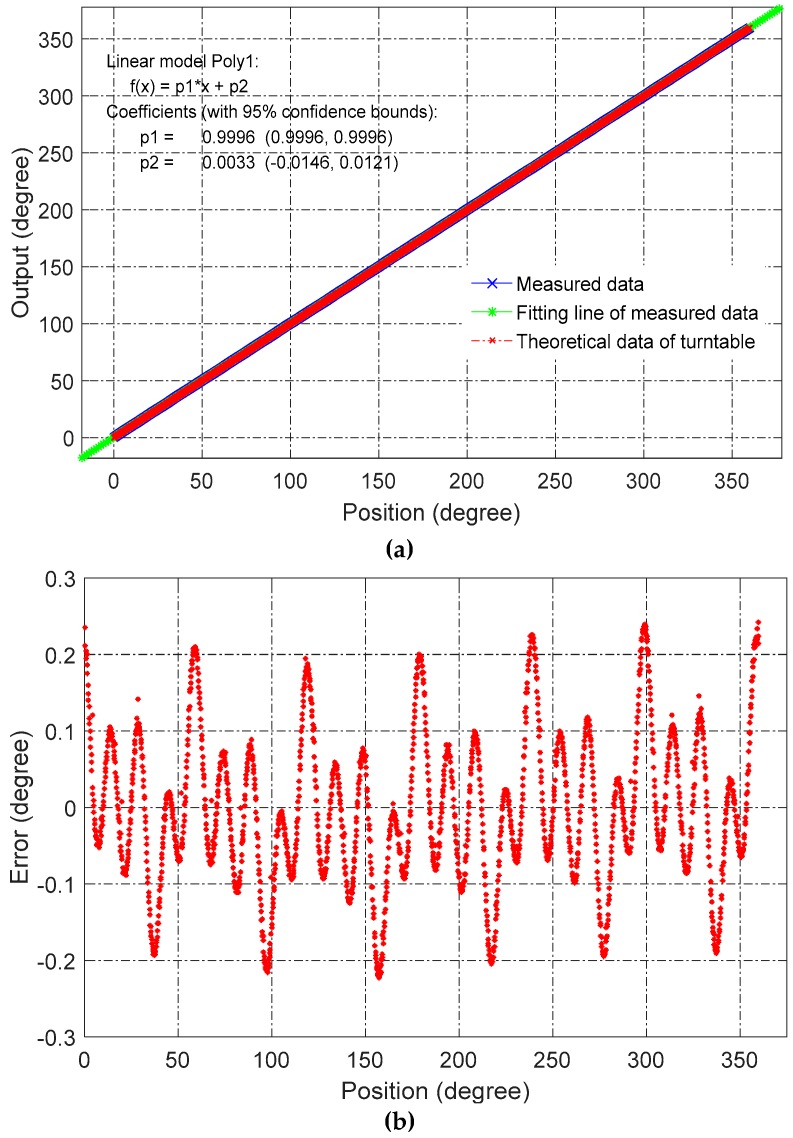
Results of improved experimental setup. (**a**) Measured angular position and best-fitting line; (**b**) nonlinearity over a full range with respect to the best-fitting line.

In an effort to reduce nonlinear error, the same prototype sensors were better assembled and tested. Further, the position between rotor and stator was finely adjusted to reduce the installation error as much as possible. As shown in Figure 12, the nonlinearity error was reduced from 0.5° to 0.25°.

### 4.2. Nonlinear Error Compensation

**Figure 13:**
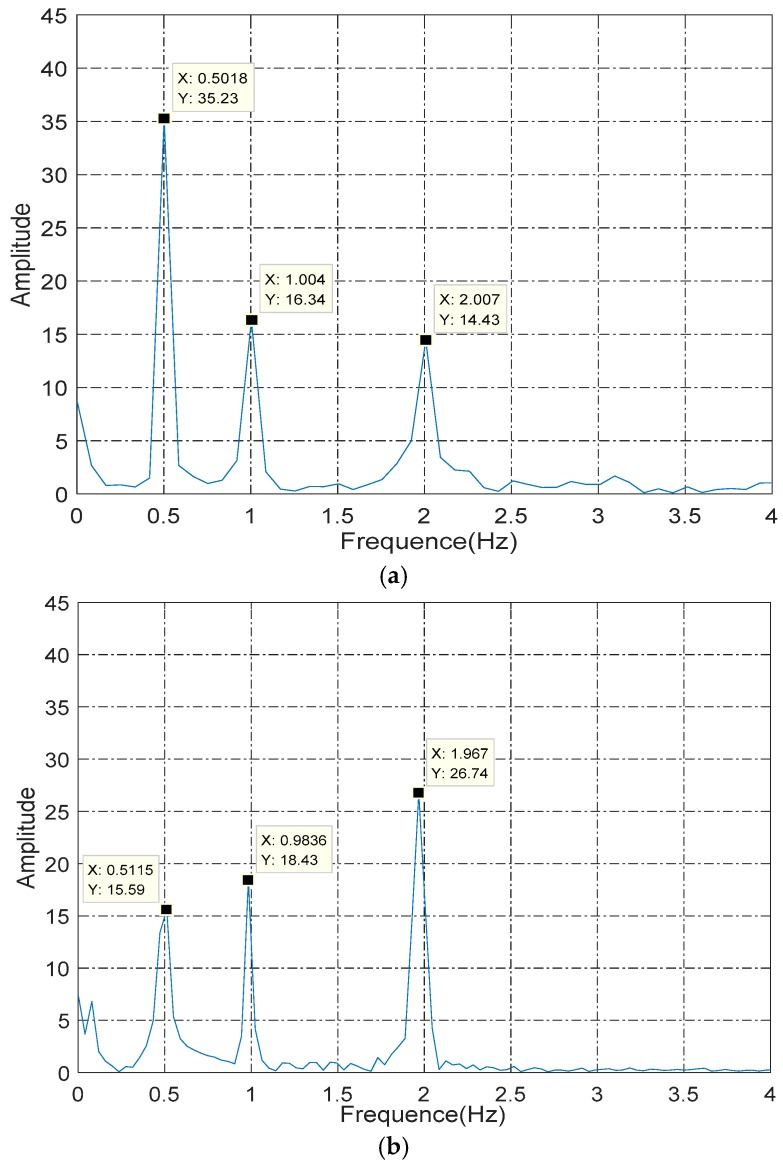
(**a**) Spectral analysis of nonlinear error before adjustment; (**b**) spectral analysis of nonlinearity error after adjustment.

After being finely adjusted, the nonlinear error of the improved prototype was reduced to 0.25°. The main components of the nonlinear error were analyzed through the FFT algorithm by the MATLAB software. Figure 13a shows the spectrum of nonlinearity before being finely adjust when the rotor rotated at 30°/s, and Figure 13b shows the spectrum after being finely adjusted. As shown, the amplitude of the frequency of 0.5 Hz (which was the main component of nonlinear error) was reduced from 35.23 dB to 23.39 dB after adjustment, and the nonlinearity error was reduced from 0.5° to 0.25°. If a better experimental setup is built to assess further, the performance of the capacitive angular sensor should be improved further. In the course of the research, a series of experiments has been conducted to try to remove the three periodic components, as shown in Figure 13, but failed (which might be because of manufacturing tolerances), which means the nonlinear error is hard to eliminate through adjustment. Therefore, the improvement to the nonlinearity through adjustment was limited, and we try to compensate the error.

Based on the spectral analysis, Equation (16) is applied to compensate the nonlinearity of CAPS,

(16) $$\theta_{R}=\theta +(A\cos(\theta_{T})+B\cos(2\theta_{T})+C\cos(4\theta_{T}))$$

where $\theta_{R}$ is the compensated angle, θ is the measured angle, A, B and C are the coefficients, which can be obtained by the least squares method, and $\theta_{T}$ is the truthful angle of the rotor, and it is obtained through the high-precision turntable. 

**Figure 14:**
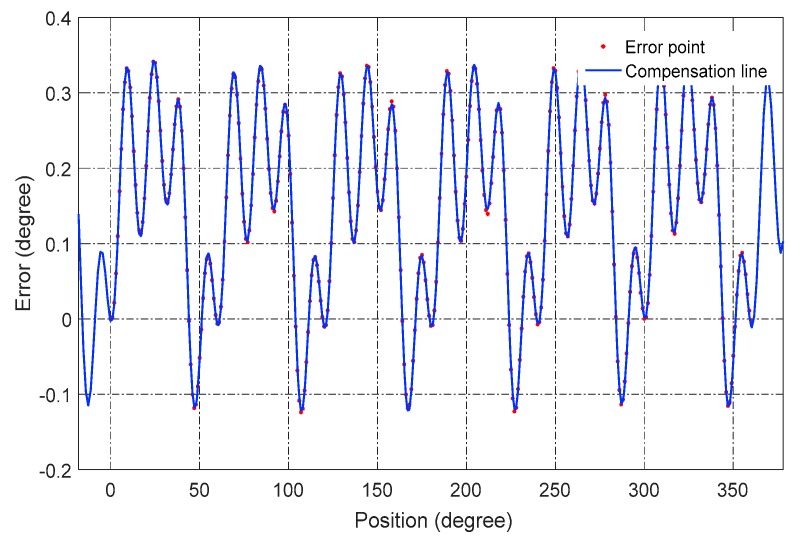
Nonlinearity compensation line.

**Figure 15:**
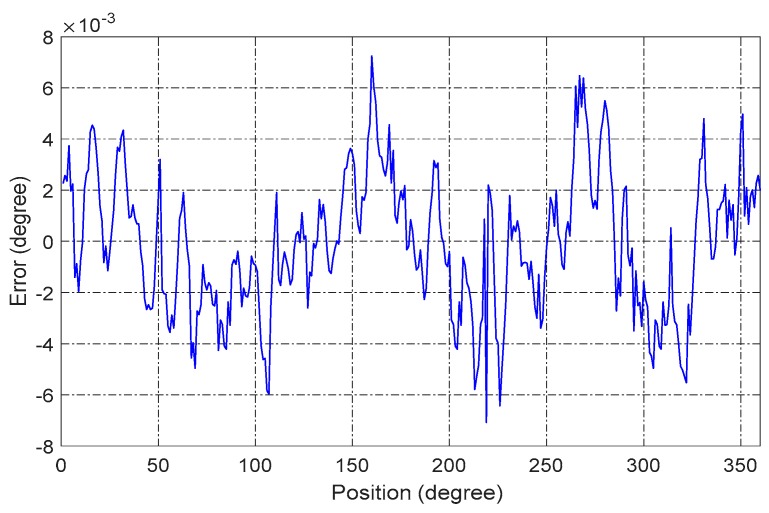
Nonlinearity error after compensation.

Figure 14 shows the nonlinearity of a full range and the compensation line to the error. The results after compensation are shown in Figure 15. The nonlinear error of the sensor is reduced to less than 0.008° over a full range, which indicates that the CAPS has great potential to be applied in high-precision applications with a low cost. The compensation for several different assembly errors has also been conducted on the mentioned method, and the result is very consistent with the present one. In a good test environment, the nonlinearity is even better than 0.008° over a full range.

## 5. Conclusions

A novel capacitive angular position sensor (CAPS) excited by single excitation voltage is presented in this paper. The CAPS encodes the angular position into amplitude-modulated signals based on the designed novel structure. The amplitude-modulated signals are then digitally decoded to angular position based on the resolver demodulation algorithm. The measurement results show that the accuracy and resolution are 0.0036° and 0.0009°, respectively. After compensation, the nonlinearity is better than 0.008° over a full range. This indicates that the proposed CAPS has significant potential to be applied for high-precision measurement.

To further assess and improve the performance of the CAPS, better experimental conditions should be designed, including using a better fabrication technology and building a housing for interference shielding. The stationary nonlinearity and more dynamical performance will be further evaluated. Future developments will concentrate on increasing the signal to noise ratio of CAPS, analyzing the fringing field effect and designing the absolute measurement method.

## Figures and Tables

**Table 1:** Key parameters of the capacitance angular sensor.

Parameter (Symbol)	Value	Parameter (Symbol)	Value
Outer diameter ($R_{1}$)	54 mm	Excitation coefficients (*k*)	0.5
Inner diameter ($R_{2}$)	27 mm	Charge amplifier ($G_{c}$)	1
Coupling capacitance ($C_{0}$)	8 pF	Dielectric constant (ε)	1.2
Width of the sensitive electrode (2τ)	8 mm	Excitation frequency (*w*)	10 kHz
Sensitive electrode diameter (*R*)	44 mm	Plate spacing (d)	1 mm
Cycle number of the sine wave (*N*)	6	Differential amplifier ($G_{a}$)	2
